# Sound effects on body perception vary with the social support network of individuals

**DOI:** 10.1016/j.isci.2025.113091

**Published:** 2025-07-11

**Authors:** Amar D’Adamo, Angel Sánchez, Lize De Coster, Ana Tajadura-Jiménez

**Affiliations:** 1 Department of Computer Science and Engineering, Universidad Carlos III de Madrid, Madrid, Spain; 2 Grupo Interdisciplinar de Sistemas Complejos (GISC), Departamento de Matemáticas, Universidad Carlos III de Madrid, Madrid, Spain; 3 Instituto de Biocomputación y Física de Sistemas Complejos (BIFI), Universidad de Zaragoza, Zaragoza, Spain; 4 Department of Applied Information Technology, University of Gothenburg, Gothenburg, Sweden; 5 UCL Interaction Centre, University College London, London, UK

**Keywords:** Social sciences

## Abstract

Mental body representations are malleable and can be influenced by auditory cues. In the “Footsteps illusion,” real-time alterations of walking sounds simulate those produced by heavier or lighter bodies, affecting perceptions of body weight, speed, and gender traits, and triggering emotional, behavioral, and physiological changes. While body illusions are known to affect social attitudes, less is known about how social factors influence body perception malleability. We investigated whether social support networks modulate this malleability using the “Footsteps illusion,” given the social relevance of body weight. A total of 105 participants experienced three footstep sound conditions (heavier, lighter and control). We collected demographic, behavioral, physiological, and subjective data, along with body image and eating disorder questionnaires. Results showed that larger social support networks correlated with higher body image satisfaction and fewer eating disorder symptoms. Notably, the illusion had a stronger effect on those with smaller social networks, highlighting the moderating role of social support.

## Highlights

• 105 people were exposed to the “Footsteps illusion,” targeting perceived body weight
• They shared data on social support networks, eating habits, and body image satisfaction
• Social support networks shape body perception: larger networks weaken the illusion
• Bigger networks link to higher body image satisfaction and less eating disorders (ED) symptoms

## Graphical abstract

**Figure:**
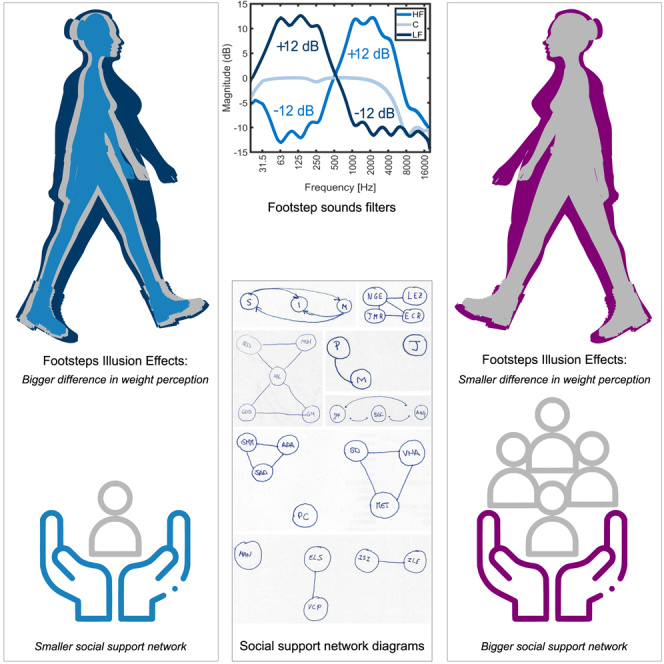

## Introduction

Our perception of our body profoundly shapes how we move, interact with others, and experience ourselves.^1^ For instance, individuals with anorexia nervosa rotate their shoulders to pass through doorways much wider than their actual shoulder width, illustrating how distorted body perception, in this case body size, influences motor behavior.^2^ These mental body representations are central to self-awareness, encompassing body ownership (i.e., the feeling that “this body is mine”) and self-identity (i.e., recognizing oneself as distinct from others),^3^^,^^4^^,^^5^^,^^6^^,^^7^ as well as the ability to find self-other equivalences (“like me”), which underpin social behaviors.^8^^,^^9^^,^^10^^,^^11^^,^^12^^,^^13^

Critically, these body representations are highly malleable, as demonstrated by body illusions induced, for instance, through immersive virtual reality, allowing individuals to experience altered body dimensions (e.g., longer arms, a shorter/taller or a slimmer/wider body, or embodying a child’s body).^14^^,^^15^^,^^16^^,^^17^ The key to these bodily illusions is the alteration of immediate bodily sensory feedback,^18^^,^^19^^,^^20^ which builds on the neural mechanisms underlying the formation of mental body representations. Neuroscientific research has revealed how multisensory integration continuously and rapidly updates mental body representations, as seen in studies using tools,^18^^,^^21^^,^^22^ rubber hands,^19^ and virtual bodies.^15^^,^^20^^,^^23^^,^^24^^,^^25^^,^^26^^,^^27^

Importantly, these body illusions, in turn, influence not only motor behavior,^14^^,^^18^^,^^21^^,^^28^^,^^29^ emotional states^29^^,^^30^ and body satisfaction,^31^^,^^32^ but also self-identity^3^^,^^4^^,^^33^ and social behavior.^9^^,^^10^^,^^11^^,^^13^ For instance, embodying avatars with outgroup physical features (e.g., different ethnicity, gender or age) can shift implicit social attitudes.^13^^,^^34^^,^^35^ In a recent study using the so-called “Footsteps illusion”^29^^,^^36^ we demonstrated that modifying one’s own footsteps sounds, to resemble those of a lighter or heavier body, changed participants’ perception of their own body weight as well as their self-perceived masculinity/femininity, supporting the idea that own body illusions shape self-perception and identity.^36^^,^^37^ Expanding on this, Clausen et al.^38^ showed that such altered footstep sounds could induce a “gender illusion,” increasing identification with one’s gender outgroup. To explain these effects of body illusions on social cognition, Maister et al.^9^ and Tsakiris et al.^39^ proposed a connection between body perception and higher-level cognition. Maister et al.^9^ specifically suggested that the mental representation of “me” integrates both a representation of the body and abstract facets of identity, such as attitudes, beliefs, and group affiliations.

While research has established that body illusions affect social attitudes toward others (see Tajadura-Jiménez et al.^15^; Peck et al.^34^; Banakou et al.^40^), research on how social factors shape the malleability of body perceptions remains limited. Thus, the two-way relationship between bodily illusions and social cognition is not yet well understood. Given that body perception shapes motor, emotional, and social functioning, as well as self-awareness, understanding this relationship is crucial for uncovering how social experiences shape our body representations. Interpersonal multisensory-stimulation experiments demonstrate that synchronous interpersonal visuotactile stimulation increases perceived physical and psychological similarity between self and others,^41^ which influences our mental self-representations. Similarly, purely social interventions—without bodily manipulation—can induce comparable effects; for instance, facial similarity between ourselves and another person, influences trustworthiness judgments and cooperative behavior, and the reverse is also true.^35^ Additionally, self-other overlap in embodied motor representations is greater between romantic partners than platonic friends,^42^ and attachment style correlates with self-other overlap at a conceptual level,^43^ showing how our social relationships influence the overlap between the mental (body) representations of ourselves and those of others.

Theoretical accounts suggest that the influence of social cognition on bodily self-representations is present from an early age, given that they are crucial for the development of the bodily self.^44^ These accounts propose that from infancy, a minimal self-characterized by a pre-reflective sense of agency^45^ and self-other distinction^46^—develops through embodied mentalization (i.e., the probabilistic representation of multisensory and sensorimotor signals) during interactions with caregivers^47^ (e.g., through mutual touch^46^). Additionally, social mediation plays a key role in bodily self-consciousness^48^ and self-agency.^49^ According to this view, we internalize the statistical regularities of our interactions, forming expectations about the world, others, and ourselves, while actively shaping the environment to align with these expectations (see also Clark et al.^50^; Varela et al.^51^). However, despite this theoretical support for the role of social interactions in shaping self-perception and mental body representations, empirical data are lacking.

**Figure 1:**
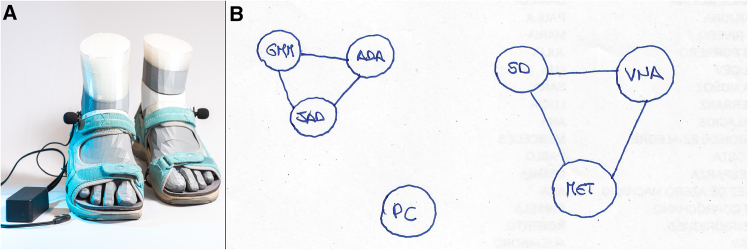
Experiment setup and tools (A) Sandals with microphones worn by the participants. Participants were asked to walk for 10 meters back and forth in a straight line for six rounds at a comfortable speed while listening to their own footsteps in three conditions differing by the applied filtering: a control condition with no filtering (C), a filter enhancing high frequencies (HF) and one enhancing low frequencies (LF). (B) An example of a social support network diagram drawn by a participant. Participants were also asked to answer the Spanish translated version of the Social Support Questionnaire (SSQ6). We obtained the following factors: #People: the number of people in the social support network, #Groups: the number of groups that compose the participants’ network, Av. Items: the average number of people in the network selected by the participant for each SSQ6 item, SD Items: the standard deviation of the number of people in the network selected for each SSQ6 item, Sex ratio: the number of people in the network that have the same sex of the participant, divided by the total number of people in the network, Age difference: the difference between the average age of the network and the participant’s age.

To address this gap, the present study investigates how social factors, more specifically our social support networks, influence the malleability of body perceptions. Specifically, we examine whether social support moderates susceptibility to the “Footsteps illusion,” focusing on body weight perception (Figure 1). Body weight is particularly relevant, as weight stigma and body ideals are deeply rooted in socio-cultural norms,^52^^,^^53^ including gender stereotypes,^33^ which drive both conscious and unconscious social comparisons. Further, prior research suggests a link between one’s social support network and body perception. For instance, social support is associated with reduced body dysmorphic disorder symptoms, with gender moderating this association.^54^ Additionally, body-specific sensory processing is automatically activated when observing others,^55^ reinforcing the idea that body image is a socially based construct tied to social factors and functioning.^56^ However, the influence of someone’s social support network on the malleability of body (weight) perception remains unclear. We hypothesize that the size and composition of social support networks modulate body weight perception shifts in the “Footsteps illusion.” By testing this, we aim to clarify whether social connections shape body perception malleability within a socially influenced own-body illusion framework.

Beyond dyadic interactions, social networks further influence health and well-being.^57^ Different network structures relate to different individual needs and serve various functions, such as buffering the effects of stress and providing emotional support and instrumental aid. As a matter of fact, community-level social cohesion has been shown to impact health outcomes.^58^ Additionally, the beneficial effects of social support networks on physical and emotional health have been demonstrated, particularly during adolescence, reducing issues in several areas of functioning in young adulthood.^59^ While positive social support can reinforce health behavior change, negative influences can undermine it.^60^ Growing research is outlining relevant pathways, including potential biological and behavioral mechanisms, allowing the design of interventions attempting to apply basic research on the positive effects of social support. It is thus important to explain why this relationship exists and the specificity of such links.^61^

## Results

### Understanding people: Social support vs. individual features

**Figure 2:**
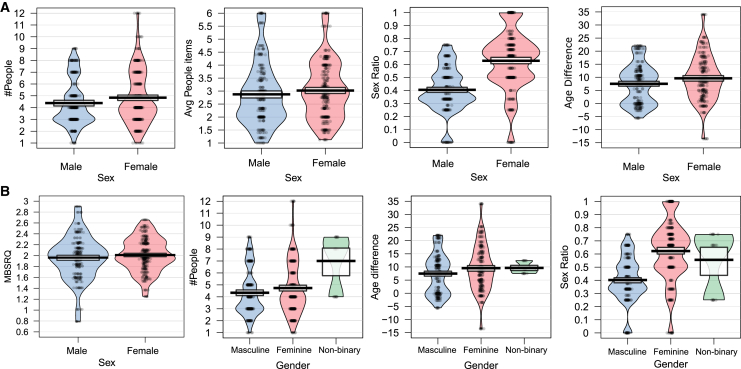
Characterization of the participants (A and B) Distributions of the social support network factors that significantly correlate with (A) Sex, (B) Gender. Questionnaire and demographic data, mean and interquartile ranges are displayed.

Let us begin the presentation of our results by discussing the interactions (sex and gender) and correlations between the basic variables characterizing the participants in the study (symptomatology of eating disorders (SED) and body image concern, characterized in terms of Eating Disorder Examination Questionnaire (EDEQ) and Multidimensional Body Self Relations Questionnaire (MBSRQ) scores) and the features of their social support networks. For the overall population, the results of non-parametric tests, specifically Mann-Whitney U tests for sex—as we have only two groups—and Kruskal-Wallis tests for gender—as we have three groups—showed significant differences between sexes and genders in relation to social support network features. For sex, we found significant differences between sexes in the number of people in the network (W = 8208, *p* < 0.001), the average number of people selected per Social Support Questionnaire (SSQ6) item (W = 9918, *p* = 0.029), the sex ratio in the network (W = 3564, *p* < 0.001), and the age difference (W = 5706, *p* = 0.012), as shown in Figure 2A). Additionally, we found significant differences between sexes in terms of MBSRQ scores (W = 9216, *p* = 0.002). Then, with respect to gender, we observed a significant interaction with the number of people in the network (W = 14598, *p* < 0.001), the sex ratio in the network (W = 19260, *p* < 0.001), and the age difference (W = 8370, *p* = 0.012), as shown in Figure 2B). Therefore, it is clear that sex, as well as gender (which is more of a social construct), are intimately related to the characteristics of the social support network, as could be expected. This result is in agreement with previous findings in the literature (see, e.g., Kneavel et al.^62^ and references therein), thus giving support to our methods and analyses.

Let us now turn to the variables that are more specific to our study. Spearman’s correlation analyses are presented in Table 1. First, when considering the EDEQ score, a significant correlation was found with the size of the network and with the average number of people selected across the questionnaire items (cf. Figure S2 in the Supplementary Material). In particular, the EDEQ score was negatively correlated with the number of people composing social support networks, meaning that the higher the level of SED, the lower the number of people composing the network. Additionally, the negative correlation with the average number of people for each item of the questionnaire shows that this is a general phenomenon and is not restricted to particular types of social support. As with the case of gender, our findings are well aligned with the literature (see, e.g., Leonidas et al.^63^ and references therein), underlining the validity of our approach.

**Table 1:** Understanding people

	EDEQ	MBSRQ	#People	#Groups	Av. Items	SD. Items	Sex Ratio
EDEQ							
MBSRQ	−0.06						
#People	−0.18∗∗∗	0.11∗∗					
#Groups	0.03	−0.10∗∗	0.30∗∗∗				
Av. Items	−0.14∗∗∗	0.04	0.14∗∗∗	−0.18∗∗∗			
SD. Items	0.02	0.01	0.18∗∗∗	0.14∗∗∗	0.09∗		
Sex Ratio	0.03	0.06	0.27∗∗∗	0.10∗	−0.17∗∗∗	0.15∗∗∗	
Age Diff.	0.08	−0.03	−0.07	−0.06	0.07	0.18∗∗∗	−0.10∗

Correlations between overall social support and individual factors of the 105 participants who took part in the study. Shown are Spearman’s correlations: ρ values and significance level—asterisks denote significant differences between means (∗∗∗ denotes *p* < 0.005, ∗∗*p* < 0.01, and ∗*p* < 0.05).

When considering the MBSRQ score, significant correlations were found with the size of social support networks. In particular, the participants’ MBSRQ score showed a positive correlation with the number of people composing the support networks; this means that a larger social support network contributes to a better body image. The MBSRQ score was shown to be negatively correlated with the number of groups (cf. Figure S3 in the Supplementary Material). There are very few studies on this aspect, making it difficult to assess our results from a general perspective. However, findings from research on a group of women with the diastasis recti abdominis medical condition seem to align with ours, identifying several predictors, (e.g., social support of partner, family, and friends) of general body appearance and health.^64^ The fact that the relationship has been demonstrated in groups of women is likely related to the correlation we also find between MBSRQ and sex (see Figure 2A).

### Interaction between social support and the effects of sound

Having characterized our participants’ key demographic features and described the relationship between their individual features and the nature of their social support, we are in a position to proceed with the study of the connections between such social support and the effects of sound on body perception.

### Questionnaire on body feelings and emotional experience

**Figure 3:**
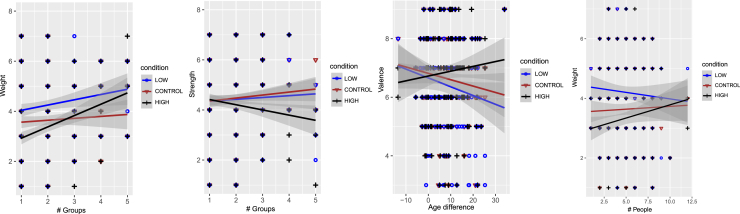
Questionnaire on Body Feelings and Emotional Experience items Interactions of the social support network factors with the Questionnaire on Body Feelings and Emotional Experience items: dots represent the observations, lines show predicted values from the model, and shaded regions represent confidence intervals. Significant effects of the number of groups and age differences in the social support network on perceived body weight, strength, and valence across sound conditions. An interaction between network size and sound on perceived body weight, approaching significance but not surviving correction for multiple comparisons, is also included due to the study’s focus on this variable.

Our first analyses concern the questionnaire on Body Feelings and Emotional Experience. It turns out that there are few interactions of social support elements with sound effects. Indeed, a significant interaction was found between the factors of sound condition and number of groups in the social support network for *body weight*, F = 4.880, *p* = 0.008, $\eta_{p}^{2}=0.045$, and *body strength*, F = 5.964, *p* = 0.003, $\eta_{p}^{2}=0.052$. As it can be observed in Figure 3, for lower numbers of groups participants’ felt body weight was modulated as expected by sound, with higher scores for the low frequency condition (LF) and lower scores for the high frequency condition (HF); but with higher numbers of groups, both HF and LF are associated with higher levels of perceived body weight as compared with the control condition (C). Further, Figure 3 also shows that for LF and C strength values increase with the number of groups, while for HF strength values decrease significantly with the number of groups. When considering the age difference between participants and people in their networks, a significant interaction was also found with the factor sound condition for *valence*, F = 5.679, *p* = 0.004, $\eta_{p}^{2}=0.063$, (participants that are older than the average age of their network felt lower valence for HF compared to LF and C, while participants that are younger compared to their support network’s average age felt significantly more valence for HF compared to LF and C). Due to the interest in perceived body weight as a variable in this study, we also report here a trend that did not survive correction for multiple comparisons, showing an interaction between the number of people in the network and the sound condition factor on perceived body weight, F = 3.177, *p* = 0.045, $\eta_{p}^{2}=0.043$. As shown in Figure 3, for smaller networks (i.e., with fewer people), participants felt lighter in the HF condition and heavier in the LF condition, consistent with the expected “Footsteps illusion” effect. However, for larger networks, the impact of sound on perceived body weight appears to diminish.

As the above summary shows, the social support network characteristic that is the most influential on body feelings is the number of groups the network contains. This number is basically the same as the amount of different social environments in which the participant’s life takes place. As shown in Figure 3, in the HF condition perceived body weight increases, while perceived body strength decreases, with increasing number of groups. The pattern observed for the small number of groups, where people feel lighter with HF and heavier with LF, is consistent with previous data on the “Footsteps illusion.” For larger numbers of groups, perceived body strength is lower in the HF condition than in the C condition, while for perceived body weight the relationship is reversed. In the LF and C conditions, we observe a moderate increase in both perceived body weight and strength with an increasing number of groups.

### Participants’ visual estimates of their own body weight

No significant interactions were found for this measure.

### Gait biomechanics

**Figure 4:**
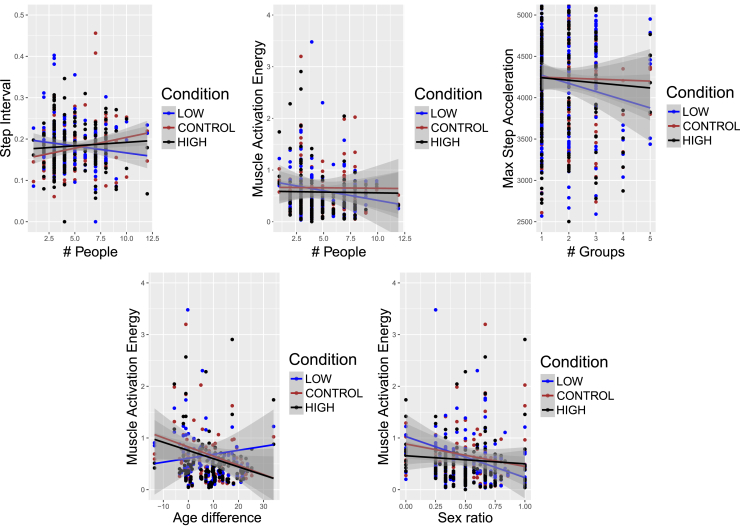
Gait Biomechanics Measures related to walking (Step Interval and Maximum Step Acceleration) and muscle (Muscle Activation Energy) activity were used as an implicit measure of changes in body perception: dots represent the observations; lines show predicted values from the model and shaded regions represent confidence intervals. Significant interactions were found between gait biomechanics features and social support network characteristics.

A significant interaction was found between the factors of sound condition and the number of people composing the support network, with two parameters related to gait biomechanics: *interstep time*, F = 6.143, *p* = 0.002, $\eta_{p}^{2}=0.063$, (higher interstep time for HF for higher number of people and lower values of interstep time for LF when the number of people in the network increases) and *electromyography (EMG) muscle activation* in terms of peak energy, F = 5.697, *p* = 0.003, $\eta_{p}^{2}=0.055$, (higher values of muscle activation energy are found for HF and C compared to LF for participants with bigger sized networks). In this context, it is important to point out that the normal trend of people walking faster with HF is reversed when the number of people in the network increases. Figure 4 summarizes the results in this subsection. As can also be seen from the plots, the factors of sound condition and the number of groups composing the support network also showed a significant interaction for *maximum foot acceleration*, F = 5.315, *p* = 0.005, $\eta_{p}^{2}=0.055$, (networks with a bigger number of groups are associated with higher values of acceleration for HF and C compared to LF). When taking into account the networks’ sex ratio, a significant interaction was found with the factor sound condition for *EMG activation energy*, F = 5.697, *p* = 0.004, $\eta_{p}^{2}=0.055$, (for participants with a network composed of people of the same sex, lower values of muscle activation energy were found: this variation is bigger for LF compared to HF and C). When considering the age difference between participants and their networks, a significant interaction was also found with the factor sound condition for *EMG activation energy*, F = 6.747, *p* = 0.001, $\eta_{p}^{2}=0.079$, (participants older than the average age of their network have higher values of muscle activation energy for HF and C compared to LF, while participants younger than their support network’s average age are associated with lower values of the muscle activation energy for HF and C compared to LF). If we now collect the findings we have just summarized, we observe that muscle activation energy is the feature that is affected by more characteristics of the social support networks, with larger changes arising in the LF condition (see Figure 4).

### Eating Disorder Examination Questionnaire score

Later in discussion, we discuss the interactions of the EDEQ score with the different variables we have obtained from the experiment. We remind the reader that a low EDEQ score corresponds to lower SED while high values of EDEQ correspond to higher SED. Plots illustrating these interactions can be found in Figure S4 of the Supplementary Material.

#### Questionnaire on body feelings and emotional experience

When adding the EDEQ score as a covariate, a significant interaction was found between sound condition, EDEQ score and number of people in the social support network for *arousal*, F = 9.824, *p* = 0.002, $\eta_{p}^{2}=0.089$, (higher EDEQ scores and number of people in the network correspond to higher levels of arousal for HF compared to LF and C). A significant interaction was also found between sound condition, EDEQ score and number of groups in the social support network for *vividness*, F = 9.382 *p* = 0.003, $\eta_{p}^{2}=0.014$, (for higher values of EDEQ and a bigger number of groups in the support network, in contrast with the overall population trend, participants felt higher levels of vividness for LF compared to HF and C). Further, we observed a significant interaction between sound condition, EDEQ score and social support networks’ sex ratio for *valence*, F = 6.246, *p* = 0.002, $\eta_{p}^{2}=0.058$, (higher levels of sex ratio correspond to lower valence for both HF and LF, for low EDEQ score. Valence in HF and LF is lower than in C, while for a high EDEQ score LF and HF correspond to higher values of valence compared to C). Moreover, a significant interaction was also found between the factors of sound condition, EDEQ score and standard deviation of people across the items of the social support networks’ questionnaire (SDPI) for *arousal*, F = 4.877, *p* = 0.008, $\eta_{p}^{2}=0.003$, (for high values of EDEQ and SDPI, participants felt higher levels of arousal for HF compared to LF and C), *body weight*, F = 5.572, *p* = 0.004, $\eta_{p}^{2}=0.052$, (for higher values of EDEQ participants felt lighter in all conditions; with increasing SDPI values participants felt less lighter in HF compared to low SDPI values, but still lighter than C and LF). When considering the age difference between participants and people in their networks, a significant interaction was also found with the factor sound condition for *body strength*, F = 6.991, *p* = 0.001, $\eta_{p}^{2}=0.078$, (participants with high scores of EDEQ that are older than the average age of their network felt higher strength for HF and LF compared to C).

#### Gait biomechanics

A significant interaction between the factors of sound condition EDEQ score and number of people in the social support networks was found for *maximum foot acceleration*, F = 6.472, *p* = 0.001, $\eta_{p}^{2}=0.067$, (higher EDEQ scores and a larger number of people in the network are associated with lower values of maximum foot acceleration for all conditions, but these values are higher for HF, compared to LF and C).

We can summarize our findings about EDEQ and its interactions as follows. Higher EDEQ scores and a larger support network lead to increased arousal for HF over LF and C, but, contrary to the general trend, they also result in greater vividness for LF compared to HF and C. Higher EDEQ scores and a bigger SDPI also lead to increased arousal for HF over LF and C, and to feeling lighter overall, with higher SDPI values mitigating the feeling in HF compared to low SDPI values, but still lighter than C and LF. Low EDEQ scores and a higher sex ratio are associated with lower valence for both HF and LF. Finally, high EDEQ scores and high age difference correspond to higher values of strength for both the manipulated sound conditions, compared to C.

### Multidimensional Body Self Relations Questionnaire score

We now present the interactions of the MBSRQ score with the experimental variables. The significant cases are collected in Figure S5 of the Supplementary Material.

#### Questionnaire on body feelings and emotional experience

When adding the MBSRQ score as a covariate (we remind the reader that a low MBSRQ score is related to negative body image while a high MBSRQ score corresponds to a positive body image), a significant interaction was found between the age difference between participants and their network’s age and the variables *body weight*, F = 5.045, *p* = 0.007, $\eta_{p}^{2}=0.057$, (increasing MBSRQ and age difference provokes a bigger difference in the perceived weight between LF and HF), *body straightness*, F = 9.092, *p* < 0.001, $\eta_{p}^{2}=0.099$, (higher values of MBSRQ and age difference correspond to higher straightness for HF compared to LF and C) and *femininity*, F = 8.300, *p* < 0.001, $\eta_{p}^{2}=0.091$ (higher values of age difference correspond to higher values of femininity for HF compared to LF and C; lower values of MBSRQ increase the difference in perceived femininity between HF, LF and C). We note that the MBSRQ did not show any other significant interactions with the factor sound condition beyond the ones related to the questionnaire on Body Feelings and Emotional Experience.

### Sex

#### Questionnaire on body feelings and emotional experience

As for the interactions between the sex variable and the experimental variables, we also found significant cases, as shown in Figure S6 of the Supplementary Material. In particular, when adding sex as a between-subjects analysis factor, a significant interaction was found between the factors of sound condition, sex and number of people in the networks for *felt walking speed*, F = 5.705, *p* = 0.004, $\eta_{p}^{2}=0.053$, (for higher numbers of people in participants’ networks, females felt quicker in HF compared to C and LF, while males felt quicker in LF and C compared to HF). Additionally, a significant interaction was found between the factors of sound condition, sex and age difference for *body straightness*, F = 6.766, *p* = 0.002, $\eta_{p}^{2}=0.076$, (for high values of age difference, female participants felt higher values of straightness for HF and C compared to LF, while male participants felt more straight-up for HF and LF compared to C).

#### Gait biomechanics

A significant interaction was found between the factors of sound condition, sex and number of people in the support network for *maximum foot acceleration*, F = 8.632, *p* < 0.001, $\eta_{p}^{2}=0.088$, (for higher numbers of people in participants’ networks: female participants accelerated more in HF and C compared to LF, while male participants accelerated more in LF compared to HF and C).

### Gender

#### Questionnaire on body feelings and emotional experience

Due to its more psychological character, the variable gender plays a more important role than sex in our study. We observed a few interactions between this variable and the other factors (cf. Figure S7 in the Supplementary Material). In particular, when adding gender as a between-subjects analysis factor, a significant interaction was found between sound condition, gender and age difference for *body straightness*, F = 3.859, *p* = 0.005, $\eta_{p}^{2}=0.088$, (for participants younger than their network’s age: more feminine participants felt higher levels of straightness in HF and C compared to LF, while more masculine participants felt higher levels of straightness in HF and LF compared to C), and for *arousal*, F = 4.057, *p* = 0.003, $\eta_{p}^{2}=0.093$, (for participants younger than their network’s age: more feminine participants felt higher levels of arousal in LF compared to C and HF, while more masculine participants felt higher levels of arousal in HF compared to C and LF).

Additionally, a significant interaction was also found between sound condition, gender and the SDPI score for *arousal*, F = 3.751, *p* = 0.005, $\eta_{p}^{2}=0.071$, (for participants with high SDPI: more feminine participants felt less aroused in HF compared to C and LF, while masculine participants felt more aroused in HF compared to C and LF).

### Big Five Personality Inventory-10

After conducting the analyses described so far, we considered the possibility that the reported interactions might be mediated by psychological factors, more specifically, certain characteristics from the Big Five framework. The rationale behind the new phase of data collection, aimed at gathering Big Five data from participants, was based on existing evidence linking some Big Five traits, particularly neuroticism, to social support networks and various emotional and mental processes.^65^^,^^66^^,^^67^^,^^68^^,^^69^ Additionally, previous studies have also reported connections between Big Five characteristics and body malleability.^70^ To explore this, we contacted participants and asked them to complete the Brief Version of the Big Five Personality Inventory (BFI-10) test via email. A total of 56 participants—roughly half of the sample—responded. We then used Spearman’s correlations to analyze relationships between the BFI-10 characteristics, SSQ6 item scores, and other individual factors (EDEQ, MBSRQ). As shown in Table 2, there is little to no correlation between the measured traits and individual differences among participants. If correlations had been found between certain Big Five traits and our experimental variables, it could suggest that the observed interactions arose from individual personality traits rather than the factors we originally focused on. However, we did not find such correlations, indicating that the interactions found between social support networks and bodily manipulations are not mediated by these personality traits. Additionally, we conducted ANCOVAs with the within-subject factors of sound condition (HF, LF, C) and repetition (1, 2), using the BFI-10 items as covariates. Based on previous literature identifying neuroticism as a potential mediator,^66^ we conducted a dedicated mediation analysis (see Table S2 in Supplementary Material) along with complementary Bayesian analyses focusing on the neuroticism dimension of the BFI-10. The analyses revealed no significant correlations between neuroticism and the social support dimensions or population individual differences, as shown in Table 3 (see also Table 2 for Bayes factors), and no statistically significant effect of neuroticism on the questionnaire items, as shown in Table 4. Both frequentist and Bayesian results consistently indicated that personality traits did not mediate the interactions between social support networks and bodily manipulation. Moreover, the Bayesian analyses provided moderate to strong evidence in favor of the null hypothesis, reinforcing the conclusion that no substantive mediation effect was present.

**Table 2:** BFI-10 Spearman’s correlations

	(1)	(2)	(3)	(4)	(5)	(6)	(7)	(8)	(9)	(10)	(11)
(1) Extraversion											
(2) Agreeableness	0										
(3) Conscientiousness	0.11	0.04									
(4) Neuroticism	0.08	−0.25	0								
(5) Openness to Experience	−0.10	−0.11	0.07	0.12							
(6) #People	0.06	−0.03	−0.02	0.11	0.27						
(7) #Groups	−0.10	−0.13	−0.05	0.27	0.06	0.27					
(8) Age difference	0.10	0.08	0.09	0.06	0	−0.06	0.07				
(9) Sex ratio	−0.05	−0.28∗	−0.01	0.22	−0.05	0.38∗∗	0.12	−0.28			
(10) MBSRQ	0.08	0.01	−0.01	−0.14	−0.01	0.25	−0.18	−0.11	0.12		
(11) EDEQ	−0.24	−0.14	−0.07	−0.04	0.15	−0.22	0.08	0.10	0.01	−0.24	

BFI-10 Spearman’s correlations (ρ values and significance) with social support dimensions and population individual differences (∗∗ denotes *p* < 0.01 and ∗*p* < 0.05).

**Table 3:** BFI-10 Bayesian correlations results

Measure	$BF_{01}$	$BF_{10}$
EDEQ	7.402	0.135
MBSRQ	3.152	0.317
#People	4.312	0.232
#Groups	5.388	0.186
SD Items	2.974	0.336
Av. Items	7.551	0.132
Sex Ratio	3.232	0.309

Bayesian correlations (${\text{BF}}_{01}$ and ${\text{BF}}_{10}$) between BFI-10 traits—focusing on neuroticism—and social support dimensions, as well as population individual differences.

**Table 4:** BFI-10 interaction with the Questionnaire on Body Feelings and Emotional Experience results

Measure	F	*p*-value	BF_01_	BF_01_
Valence	0.033	0.967	3.717	0.269
Arousal	0.123	0.884	9.615	0.104
Dominance	0.412	0.663	9.009	0.101
Felt walking speed	0.301	0.741	5.128	0.195
Weight	0.896	0.411	5.128	0.192
Strength	0.002	0.998	18.518	0.054
Straightness	0.725	0.487	2.994	0.334
Femininity	0.024	0.976	4.587	0.218
Proprioception	0.024	0.976	3.906	0.256
Vividness	2.331	0.103	16.129	0.062
Surprise	2.642	0.076	3.058	0.327
Agency	1.114	0.332	125.000	0.008

ANOVAs and Bayesian analysis results (${\text{BF}}_{01}$ and ${\text{BF}}_{10}$) for neuroticism BFI-10 item.

## Discussion

### Results overview

In this study we investigated whether social support networks influence the malleability of body perception through a sound-driven illusion, the “Footsteps illusion,” where real-time alterations in footstep sounds create the perception of having a heavier (low frequency sound condition) or lighter (high frequency sound condition) body. By targeting perceived body weight, this illusion is especially relevant to this context, given the social stigmas associated with weight.^52^^,^^53^ Our findings reveal significant interactions between social support variables and body perception malleability, both on the overall population as well as based on individual differences. First, our results show that the number of people and groups in one’s social support network emerges as a crucial determinant of the malleability of body perception. Notably, these social variables modulate the impact of the different sound conditions on perceived body weight, the primary target of the “Footsteps illusion.” For participants with a lower number of people and groups in their social support networks, perceived body weight responded as expected to sounds cues (i.e., higher perceived weight for LF and lower for HF), as reflected in questionnaire responses and gait patterns (e.g., step interval, acceleration). However, larger social networks (in terms of both people and groups) appeared to weaken, or counteract, the influence of sound cues on perceived body weight. Additionally, we found significant interactions between the number of groups and sound conditions on perceived strength, as well as interactions involving other social support variables and sound effects, which deserve further investigation. Second, individual characteristics interacted with social variables when looking at the effects of sound conditions on body feelings and emotion. For instance, participants’ felt body weight, as well as arousal, were influenced by interactions between SED (reflected in EDEQ scores), sound condition and the standard deviation of people across items in the social support networks questionnaire. EDEQ scores also interacted with other parameters of the social support network to influence participants’ felt body straightness, vividness of the feelings about the body, and emotional valence across different sound manipulations. Similarly, we observed how sound effects on the different variables of interest were modulated by the interaction of body image concerns (reflected in MBSRQ scores) with people’s age difference relative to their network’s age. Additionally, gender differences significantly affected responses to sound conditions within social contexts: younger participants relative to their network’s age showed distinct patterns in arousal and body straightness across different sound manipulations. These triple interactions between sound conditions, social support network variables and body concerns/satisfaction (EDEQ and MBSRQ scores) highlight the importance of taking individual differences into account when looking at the relationship between social support variables and the malleability of body perception, and these complex relationships require further study.

While previous research has linked social support networks to body perception^71^ and overall physical and mental health,^72^^,^^73^ the mechanisms underlying these effects remain unclear. In this context, our results suggest that social support networks provide a figurative “armor” against the influence of external bodily cues on our mental body representations. People with fewer individuals and fewer groups in their networks showed changes in body weight perception in the direction suggested by the sound cues. In contrast, those with larger, more diverse networks appeared less affected by these external signals or responded in a way that diverged from what the sound cues indicated. Several interpretations of this “armor” phenomenon can be considered. A first interpretation relates to exposure to diverse body ideals: larger or more diverse social networks may expose individuals to a broader range of body types and perspectives, potentially leading to less rigid body standards. Conversely, smaller or more homogeneous networks may reinforce specific body ideals, possibly leading to body dissatisfaction and SED, as reflected in the correlations we observed between network size, EDEQ and MBSRQ scores. Further, feeling connected and accepted within a social group is crucial for psychological well-being.^57^ Strong social connections can foster a sense of belonging and positive self-identity, which, in turn, may contribute to a healthier body image and a lower risk of SED.^63^^,^^74^ Our result that the number of social groups influences the malleability of body perception further supports this idea, by suggesting that a larger social network leads to a more stable (and possibly healthy) body image. Another interpretation touches upon the influence of external signals on body perception as generators of stress and negative emotions associated with body image concerns—factors often linked to body image dissatisfaction and SED. Individuals with larger and more diverse support networks may develop better coping mechanisms against the influence of external cues on their body image. In any event, our results are in line with, and reinforce, recent theoretical accounts^6^ that postulate that social interactions form the basis for the dynamic development of our bodily self and mental body representations.

### Takeaways

Another possible reason for the observed relationship between social support and body perception lies in the concept of body image, which consists of two key components.^75^ The perceptual body image refers to the accuracy in perceiving one’s own body size and shape, while the attitudinal body image encompasses one’s feelings and thoughts toward the shape and size of one’s own body^76^^,^^77^ (see review Cornelissen and Tovée^78^). The perceptual body image is continuously updated in response to the inputs that we receive from our body (i.e., visual, auditory, or haptic signals, among others).^79^^,^^80^ Research in multisensory integration has provided insights into the processes underlying this perceptual component,^18^^,^^19^^,^^20^ also shedding light on perceptual disturbances in body image^79^ that might be a contributing factor in the onset, maintenance, and relapse of SED.^81^^,^^82^ Notably, body image malleability and overall mental body representations have been widely studied using multisensory body illusions.^19^^,^^29^ Attitudinal body image, on the other hand, is the primary focus of cognitive-behavioral therapies aimed at addressing body image disturbances.^83^ Since body image is socially constructed^84^ and shaped by sociocultural influences such as ideal body images,^52^ body types^53^ and gender stereotypes, it is likely that social support primarily affects this attitudinal component of body image. This, in turn, may exert a top-down influence on body perception. Indeed, as suggested by,^29^^,^^36^^,^^85^ (body) perception involves predictive processes that continuously compare bottom-up signals and top-down predictions. Supporting this idea, Mussap and Salton in^86^ used the Rubber Hand Illusion^19^ to explore the relationship between body image perception and SED. They found a significant relationship between SED and the susceptibility to the illusion. Further, Tajadura-Jimenez, Crucianelli et al. in^85^ applied the “Footsteps illusion” paradigm to individuals with High SED and anorexia nervosa. In HF, which typically induces the perception of a lighter body, these participants instead visualized their body as wider and adjusted their gait to reflect a heavier body. This suggests disturbances in sensory integration mechanisms related to SED, likely due to a conflict between external bodily cues and attitudinal and perceptual expectations about their own body being heavy. Our findings align with this perspective, by suggesting that a larger social support network may reduce sensitivity to external bodily cues, potentially decreasing body perception malleability because of a more stable, healthy body image. Remarkably, for a larger number of groups, we observe that both the HF and LF conditions diverge from the Control condition in the same direction, rather than in opposite directions as suggested by the sound cues. In this context, the social support network might have an influence on the attitudinal components of body perception, which then influences the perceptual body image via top-down processes that modulate multisensory integration. It has to be noted, however, that the relationship between perceptual and attitudinal body image remains debated, mainly because it is unclear whether these two facets of body image are independent or strongly linked to each other.^81^^,^^86^ Thus, further research is needed to distinguish these facets and explore how social support networks influence body representation malleability.

Additionally, our prior work^4^^,^^87^ suggests that, beyond multisensory integration, interoceptive sensitivity—the ability to perceive internal bodily signals—also plays a crucial role in body representation. Specifically, in studies using the “enfacement” illusion and the rubber-hand illusion, we found that individuals with low interoceptive sensitivity were more susceptible to multisensory illusions, indicating a greater reliance on external bodily cues. These findings support the view that interoceptive predictive coding models are used to monitor and assign the sources of sensory input either to the self or to others, i.e., controlling the involvement of our social relationships.^4^^,^^87^ This raises an interesting possibility that should be further investigated in future works: the size of an individual’s social support network may influence body perception malleability by modulating sensitivity to internal versus external bodily cues.

### Future work

It is clear that further work is needed to assess the role of the above mentioned possibilities in understanding the interaction between our social support networks and our body perception and its malleability. A deeper look into individuals’ key personality features through specifically designed experiments would provide invaluable information to prove or disprove any role of neuroticism or other personality characteristics. Similarly, future work should evaluate whether interoceptive awareness/sensibility is higher with a larger social network, to assess whether this leads to becoming better at reading internal cues vs. becoming influenced by external factors. This further investigation may include interoceptive perception tests, such as the heartbeat detection task and interoceptive questionnaires.^88^

### Conclusion

Our findings highlight the critical role of social support networks in shaping the malleability of body weight perception, underscoring the socially rooted and dynamic nature of mental body representations. By demonstrating that social connections influence susceptibility to the “Footsteps illusion,” this study provides new insights into the interplay between social factors and body perception, contributing to a deeper understanding of how sociocultural norms and interpersonal relationships modulate embodied experiences and self-perception. This research underlines the need to further investigate the mechanisms underlying the influence of social support networks on the malleability of body perception. It also paves the way for future investigations into socially driven interventions aimed at fostering positive body image and mitigating the impact of weight stigma and body-related biases.

### Limitations of the study

While we have shown that there are several significant effects between the social network parameters of individuals and their body perception, the mechanisms underlying these effects require further study. In our analysis above we proposed a first explanation in terms of personality features, and in particular of neuroticism (i.e., neuroticism mediates the effect of social support variables on body perception malleability), but our data do not support this hypothesis, albeit with the caveat that our sample of Big Five data is relatively small and better statistics are needed. The relatively low statistical power for our Big Five analyses is partly due to the fact that only approximately half of the study participants completed the BFI-10 assessment. Post-hoc power analyses indicated that these analyses were underpowered, limiting our ability to draw definitive conclusions. As a result, we emphasize that these findings should be interpreted with caution, and we cannot exclude the possibility that personality traits may play a role in (partially) explaining our results, thought we found moderate to strong evidence that no substantive mediation effect of neuroticism was present. Future studies with larger, well-powered samples are needed to further investigate these potential associations. Finally, we acknowledge that the questionnaire on Body Feelings and Emotional Experience used in the present study, while previously employed in research, has not undergone formal validation. Nevertheless, the Spanish version of this questionnaire has been used in prior studies conducted in Spain.^85^^,^^89^ Moreover, an adapted and extended version has been applied to populations with low levels of physical activity,^90^ as well as to healthy populations.^91^ Despite its prior use, the lack of formal validation presents a potential limitation that should be considered when interpreting the results.

## Resource availability

### Lead contact

Further information and requests for resources should be directed to and will be fulfilled by the lead contact, Ana Tajadura-Jiménez (atajadur@inf.uc3m.es).

### Materials availability

This study did not generate new materials.

### Data and code availability

All subjective reports and gait data have been deposited at Mendeley Data and are publicly available as of the date of publication. Accession numbers are listed in the key resources table. This article does not report original code. Any additional information required to reanalyze the data reported in this article is available from the lead contact upon request.

## Acknowledgments

We would like to thank Lara Maister for referring us to relevant related work. This research was supported by the 10.13039/501100000781European Research Council (ERC) under the European Union’s Horizon 2020 research and innovation programme (grant agreement No 101002711; project BODYinTRANSIT) and by the PID2023-150259OB-C21 project funded by MICIU/AEI (10.13039/501100011033) and the European Union.

A.S. acknowledges financial support from the PID2022-141802NB-I00 (BASIC) grant funded by MCIN/AEI and by “ERDF A way of making Europe,” and from grant MapCDPerNets—Programa Fundamentos de la Fundación BBVA 2022. L.D.C. was supported by the CONEX-Plus programme (No. 801538) funded by 10.13039/501100006318Carlos III University of Madrid and the European Commission through the Marie Sklodowska-Curie COFUND Action (H2020-MSCA-COFUND-2017-GA801538).

## Author contributions

Conceptualization, A.D., A.S., and A.T.; investigation and data curation, A.D.; funding acquisition, project administration, and supervision, A.T.; formal analysis, A.D., L.D.C., and A.T.; writing - original draft, writing - review and editing, A.D., A.S., L.D.C., and A.T.

## Declaration of interests

The authors declare no competing interests.

## STAR★Methods

### Key resources table

**Table:** 

REAGENT or RESOURCE	SOURCE	IDENTIFIER
**Deposited data**		

Subjective reports and gait data	Mendeley Data	https://doi.org/10.17632/2v5pg8xybh.1

**Software and algorithms**		

MATLAB version rR2022a	Mathworks	https://uk.mathworks.com/products/matlab.html
RStudio version 2023.06.1 Build 524	Posit Software, PBC	https://posit.co/download/rstudio-desktop/
R version 4.3.1	R Core Team	https://www.r-project.org/
JASP version 0.19.3	JASP	https://jasp-stats.org/

### Experimental model and study participant details

#### Participants

105 healthy participants (mean age ± SD: 25.01 ± 7.20 years, range: 18–53; Sex: 47 males and 58 females; Gender: 45 masculine, two non-binary, one non-declared and 57 feminine), naive to the study aim were recruited through the Carlos III University of Madrid participant pool, public advertisements, social networking sites, and flyers. They were pre-screened for symptomatology of eating disorders (SED) using the Spanish version of the Eating Disorder Examination Questionnaire (EDEQ),^92^ which has been validated and for which normative data has been provided in Spanish-speaking countries.^93^ We note that lower EDEQ scores indicate lower SED, while higher scores of EDEQ correspond to higher SED. Participants were also pre-screened for physical activity (PA) levels using the Spanish version of the International Physical Activity Questionnaire (IPAQ),^94^ validated in Spanish.^95^^,^^96^ However, only EDEQ data is reported in the present study (for analyses using the IPAQ scores, see D’Adamo et al.^37^).

Participants provided the following information during recruitment: demographics, including age, sex (man, woman, non-binary, decline to state), gender (masculine, feminine, non-binary person, decline to state); professional experience; and anthropometric measurements (height, weight, waist and hip circumferences). This information was used to select the appropriate size for the motion tracksuit used in the study. Later, during the study session, the experimenter directly measured the participants’ anthropometric data. These values are reported in Table S1. Participants were also asked to answer the Spanish translated version^97^ of the Social Support Questionnaire (SSQ6^98^), as employed in Friedman et al.^99^ Specifically, they provided the initials of up to nine individuals in their social support network for each of the following items.

(1) Who can you count on when you need help?
(2) Whom can you really count on to help you feel more relaxed when you are under pressure or tense?
(3) Who accepts you totally, including both your worst and your best points?
(4) Who can you really count on to care about you, regardless of what is happening to you?
(5) Whom can you really count on to help you feel better when you are feeling generally down in the dumps?
(6) Who can you count on to console you when you are very upset?

They also filled in a table providing information about people’s age, gender, and relationships (friends, family, no relationships if none) between the members of the network. Relationships between their nominees were represented as a diagram, see Figure 1B). Using these data on social support networks, we calculated the following variables.

(1) #People: the number of people in the social support network;
(2) #Groups: the number of groups that compose the participants’ network;
(3) Av. Items: the average number of people in the network selected by the participant for each SSQ6 item;
(4) SD Items: the standard deviation of the number of people in the network selected for each SSQ6 item;
(5) Sex ratio: the number of people in the network that have the same sex of the participant, divided by the total number of people in the network;
(6) Age difference: the difference between the average age of the network and the participant’s age.

Participants were also asked to fill in the Spanish version of the Multidimensional Body Self Relations Questionnaire (MBSRQ),^100^ for which normative values have been provided for Spanish populations,^101^ which assesses individuals’ self-attitudinal aspects toward their physical body and relates to negative/positive body image or body concerns. For the MBSRQ, a low score is associated with a negative body image, while a high score corresponds to a positive body image.

Following the results of our data analyses, and in order to seek possible explanations of the findings reported below, participants were later asked to complete the Spanish-translated version of the BFI-10 questionnaire,^102^^,^^103^ a 5-point Likert-type questionnaire (from strongly disagree to strongly agree) that has been used to assess the main individual psychological traits within the Big Five framework in research related to body illusions.^104^

The study was conducted in accordance with the ethical standards laid down in the 1964 Declaration of Helsinki and its later amendments and was approved by the Committee of Ethics in Research of Carlos III University of Madrid. All participants gave their informed consent prior to participation and received compensation for their time.

### Method details

#### Participants inclusion and exclusion criteria

Inclusion criteria included: self-reported normal hearing acuity; proficiency in Spanish language; being able to understand the study information and give consent; being able to communicate. Exclusion criteria included: being under 18 or above 70 years of age; psychiatric or neurological diagnoses; regular use of medical or recreational psychoactive drugs; disabling orthopedic or neuromuscular diseases that necessitate assistance in walking trials; any condition that might interfere with the sensors’ placement (e.g., use of cardiac pacemaker); unable to give informed consent; deafness or acute hearing conditions.

#### Apparatus and materials

A system to modify footstep sounds in real-time while participants walked was used. It is an improved version of devices from previous studies,^29^^,^^36^^,^^38^^,^^105^^,^^106^ designed for wearability, comfort and data preservation. This system, detailed in De La Prida et al.^107^ and D’Adamo et al.,^37^ consists of strap sandals with microphones to capture footstep sounds, see Figure 1A). The key difference from previous versions is the use of a DSP electronic board (Bela.io) to alter the footstep sounds, creating three conditions: “High Frequency” (HF), in which the frequency components of the footstep sounds in the range 1–4 kHz were amplified by 12 dB and those in the range 83–250 Hz attenuated by 12 dB (to make them seem produced by lighter bodies); a “Low Frequency” (LF) condition, in which the frequency components in the range 83–250 Hz were amplified by 12 dB and those above 1 kHz were attenuated by 12 dB (to make them seem produced by heavier bodies); and a “Control” (C) condition, in which participants heard their natural footsteps sounds equally amplified across frequency bands. These conditions were used in previous studies.^29^^,^^106^ Participants heard these modified sounds through headphones with high noise reduction. To measure participants’ movements, we used a Rokoko smart suit with 19 inertial measurement units (IMU). Four suit sizes were available. Participants wore a cotton T-shirt and trousers for comfort. We collected physiological data using a Bitalino PLUX system, including electromyogram (EMG), electrocardiogram (ECG) and electrodermal activity (EDA). These data are not analyzed in the present paper except for the EMG data. Respiration data was also collected; however, it was discarded due to noise.

### Quantification and statistical analysis

A within-subjects experimental design was conducted, with participants exposed to two repetitions of the three sound conditions (i.e., HF, LF, and C) presented in a randomized order. First, to better understand our population, we examined correlations between the social support parameters and the basic variables characterizing the participants (i.e., sex, gender, symptomatology of eating disorders (EDEQ) and body image concerns (MBSRQ)). We then performed interaction analyses to explore the interplay between individual social support parameters and sound conditions. For this purpose, we conducted 3 × 2 ANCOVAs with the within-subject factors being sound condition (HF, LF, C) and repetition (1, 2). The six variables emerging from the SSQ6 were entered as covariates, one at a time, resulting in six ANCOVAs. Alpha levels were set to 0.05, and *p*-values were assessed for significance using the Benjamini-Hochberg-procedure, also known as “false discovery rate” (FDR) control.^108^ This procedure was used to identify significant results while controlling for multiple comparisons. Subsequently, additional ANCOVAs with the same within-subject factors were conducted to investigate potential interaction effects of other basic variables characterizing the participants (EDEQ and body image concerns). For each of the six ANCOVAs specified above, we added a second covariate (EDEQ or MBSRQ scores). *p*-values were again assessed for significance (FDR control for multiple testing). Finally, for the variables sex and gender, each of the six ANCOVAs specified above (with the SSQ6 items as covariates), included either sex or gender as a between-subjects factor. When significant differences where found, ∗∗∗ denotes *p* < 0.005, ∗∗ denotes *p* < 0.01 and ∗*p* < 0.05. Note that, for the gender analyses, we excluded the data from the participant who did not disclose their gender. *p*-values were once more assessed for significance (FDR control for multiple testing). Statistical analyses were performed in R (version 4.3.1, R Core Team, 2018). We conducted formal tests of normality (Shapiro-Wilk), which indicated deviations from normality for some variables. However, given our factorial design (involving both factors and covariates) we opted to retain the ANOVA-based analyses to facilitate the interpretability of the results and because ANOVA is generally considered robust to moderate violations of normality,^109^ especially when group sizes are reasonably large and balanced.^110^^,^^111^ In our study, the group size was *N* = 105, and variance homogeneity was confirmed by Levene’s Test, which further supports the reliability of the ANOVA results under these conditions.

#### Measures

To assess the impact of sound conditions on participants’ experience, we used the following measures, recorded either during (physiological and motor) or after (questionnaires) the experimental manipulation. For a full description of the measures refer to D’Adamo et al.^37^

##### Questionnaire on body feelings and emotional experience

We quantified body feelings using a Likert-type questionnaire, adapted from^29^^,^^36^ and comprising 9 statements. The first 5 items assessed the felt walking speed (ranging from slow to quick), body weight (ranging from light to heavy), body strength (ranging from weak to strong), body straightness (ranging from stooped/crouched to elongated/extended) and the feeling of being more feminine/masculine. The next 4 items assessed the agreement levels (from strongly disagree to strongly agree) with statements, including one checking whether participants felt agency over the sounds, as many studies have shown that large discrepancies between modalities and delays between actions and sensory feedback disrupt agency and diminish the sensory-induced bodily illusions.^112^^,^^113^ Additional statements assessed whether participants had vivid (vividness) or unexpected (surprise) feelings about their body,^113^ and feelings on feet localization which we refer to as proprioception, as sound may interact with proprioception.^113^ Emotional experience was assessed using the self-assessment manikin questionnaire,^114^ consisting of three 9-item graphic scales of valence, arousal and dominance. This questionnaire, widely used in assessing emotional responses to acoustic stimuli,^115^ has been applied in prior studies on the “Footsteps illusion”.^29^^,^^36^^,^^105^

##### Participants’ visual estimates of their own body weight

We used the Body Visualizer tool,^116^ employed in similar studies for the same purpose.^16^^,^^29^^,^^36^^,^^85^^,^^105^ Following each experimental trial, participants performed the task twice. The experimenter set the height of the avatar to match the participants’ and set the initial weight to 75% or 125% of the participants’ actual weight. Participants then adjusted the “weight” dimension of the avatar to reflect their own perceived body size (see similar procedures^29^^,^^36^^,^^85^). The order of the initial weight (75% or 125%) was counterbalanced across two subsequent repetitions. The analysis considered the average from the two trials for each condition.

##### Gait biomechanics

These were used as an implicit measure of changes in body perception, following previous studies^29^^,^^36^ suggesting that the sound-driven illusion of altered body weight results in people adapting their gait to the prototypical motor pattern of lighter/heavier bodies. As in those studies, we quantified leg lifting acceleration and time parameters for each gait cycle (i.e., the time between two successive steps made by one foot),^117^ including stance and swing phase times. Gait patterns are characterized by larger leg accelerations for people with lighter bodies and slower gait speed and a longer duration of the stance and the heel strike for people with heavier bodies.^118^^,^^119^ Leg and shoulder muscular activity was recorded using EMG sensors on the vastus medialis oblique and cervical trapezius sites, see Figure S1, in Supplementary Material, for details on the sensor locations. Gait and physiological data were processed in MATLAB (version R2022a). We used band-pass and low-pass Butterworth filters for ECG, EDA, and EMG signals, according to the Bitalino device (Opensignals) toolkit. Leg and shoulder muscular activity was recorded using EMG sensors on the vastus medialis oblique and cervical trapezius sites, as shown in Figure S1. After filtering, for each muscle activation interval, we obtained the signal peak, mean and energy (calculated using the integral of the squared EMG signal magnitude). We measured electrodermal activity (EDA), linked to emotional arousal and stressful states (emotional sweating).^120^ Specifically we considered EDA’s tonic component, extracting also minimum, mean and maximum values. Electrocardiac activity (ECG) was analyzed to extract heart rate and the time-domain parameters of heart rate variability: SDNN (standard deviation of normal-to-normal intervals), RMSSD (root-mean-square of successive differences), and pNN50 (percentage of successive normal-to-normal intervals that differ by more than 50 milliseconds), see Shaffer and Ginsberg; Kumar et al.^121^^,^^122^ Note that these data are not analyzed in the present paper except for the EMG data.
