# Supplementary material for: Sound effects on body perception vary with the social support network of individuals
Source: iScience. 2025 Jul 11;28(8):113091. doi: 10.1016/j.isci.2025.113091 (PMC12314398; doi:10.1016/j.isci.2025.113091)
Supplement: Document S1. Figures S1–S7, Tables S1 and S2 [file mmc1.pdf]

iScience, Volume *28*

Supplemental information

Sound effects on body perception vary with the social support network of individuals

Amar D'Adamo, Angel Sánchez, Lize De Coster, and Ana Tajadura-Jiménez

Supplementary Information

0.1 Study Participants

Demographics	MEAN(SD)	Social Support Networks	MEAN(SD)
Weight (kg)	65.72(10.87)	Number of people	4.66(2.14)
Height (cm)	169.83(8.54)	Number of groups	1.84(1.03)
EDEQ score	1.29(0.93)	Average Age (of people in the network)	33.76(9.16)
MBSRQ score	1.99(0.35)	Age Difference	8.62(8.20)
		Sex Ratio	0.53(0.23)

Table S1: **Participants demographics data.** Demographic data of the N=105 participants who took part in the study.

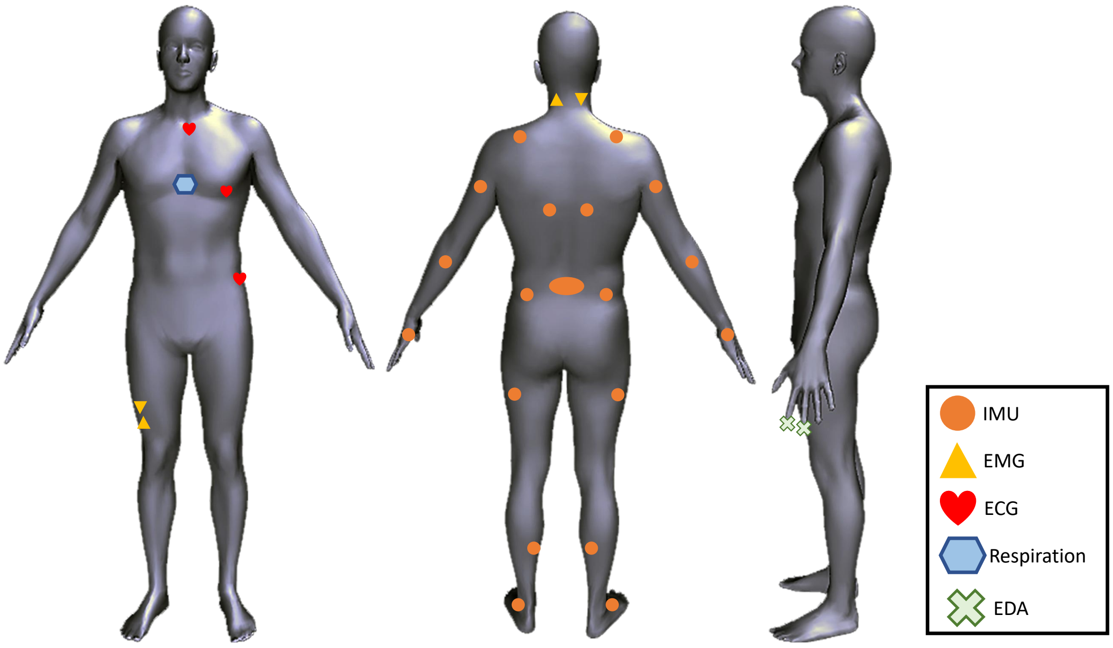

Figure S1: **Sensor locations.** IMUs, and physiological sensor locations.

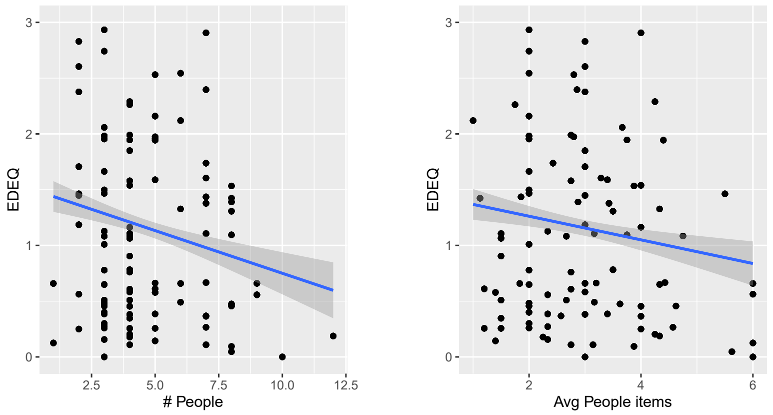

Figure S2: **Characterization of participants - EDEQ.** Significative correlations between EDEQ and social support variables. Dots represent the questionnaire data, lines show predicted values from the model and shaded regions represent confidence intervals.

**Measure**	**p (Direct)**	**p (Indirect)**	**p (Total)**
Happiness	0.995	1.000	0.995
Arousal	0.687	1.000	0.681
Dominance	0.669	1.000	0.667
Quickness	0.388	1.000	0.387
Weight	0.063	1.000	0.061
Strength	0.405	1.000	0.402
Straightness	0.409	1.000	0.407
Masculinity	0.417	1.000	0.414
Proprioception	0.115	1.000	0.113
Vividness	0.256	1.000	0.255
Surprise	0.124	1.000	0.123
Agency	0.744	1.000	0.744

Table S2: **Mediation Analysis.** Results of the mediation analysis reporting the p-values for the direct, indirect, and total effects. The mediator is neuroticism, the independent variable is sound condition, and the dependent variables are the questionnaire items on Body Feelings and Emotional Experience.

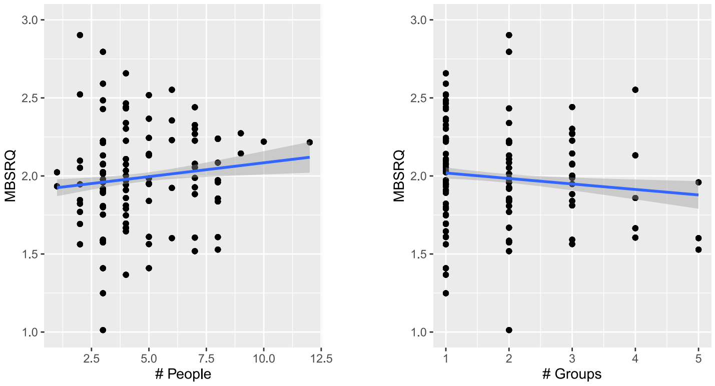

Figure S3: **Characterization of participants - MBSRQ.** Significative correlations between MBSRQ and social support variables. Dots represent the questionnaire data, lines show predicted values from the model and shaded regions represent confidence intervals.

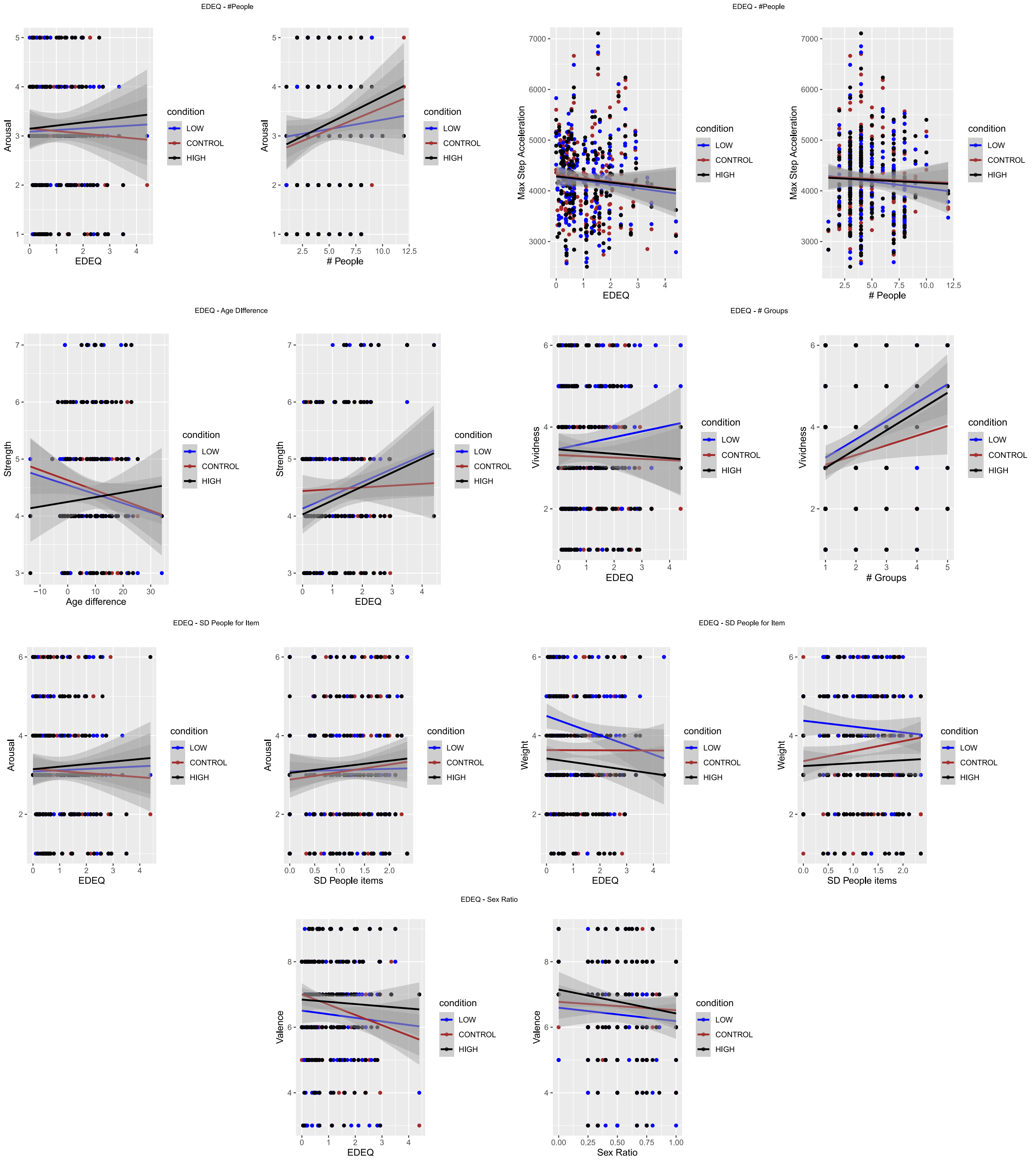

Figure S4: **Interactions with covariates.** EDEQ score. Dots represent the observations, lines show predicted values from the model and shaded regions represent confidence intervals.

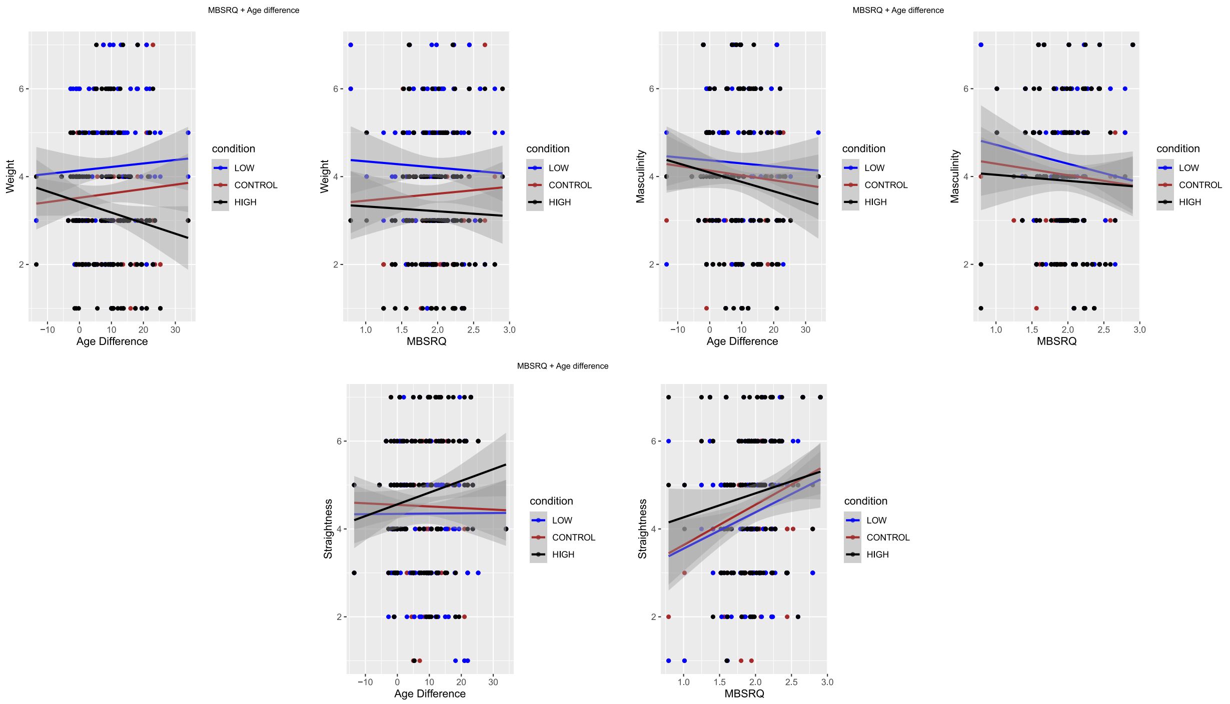

Figure S5: **Interactions with covariates.** MBSRQ score. Dots represent the observations, lines show predicted values from the model and shaded regions represent confidence intervals.

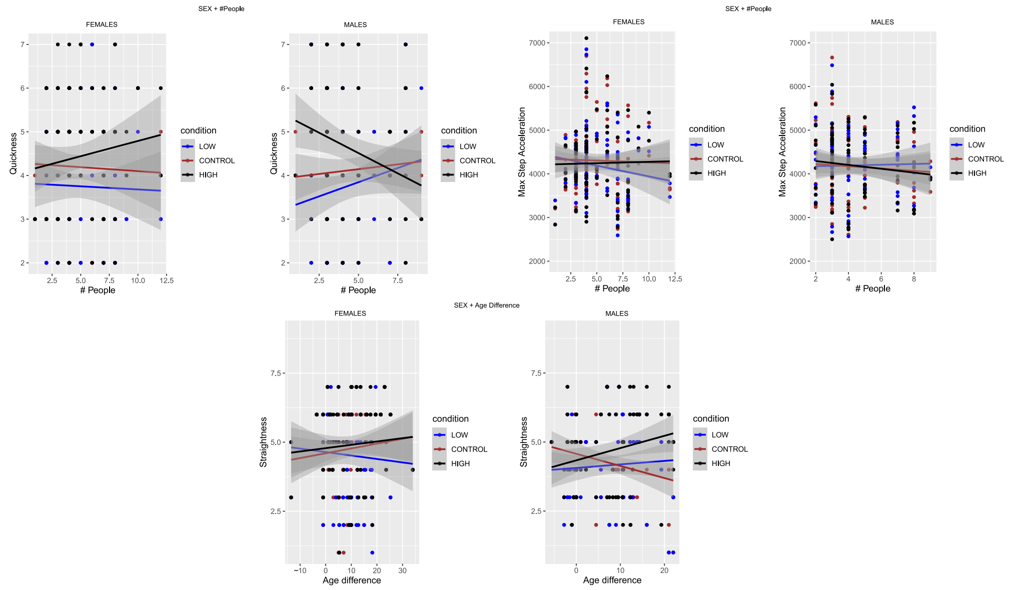

Figure S6: **Interactions with covariates.** Sex. Dots represent the observations, lines show predicted values from the model and shaded regions represent confidence intervals.

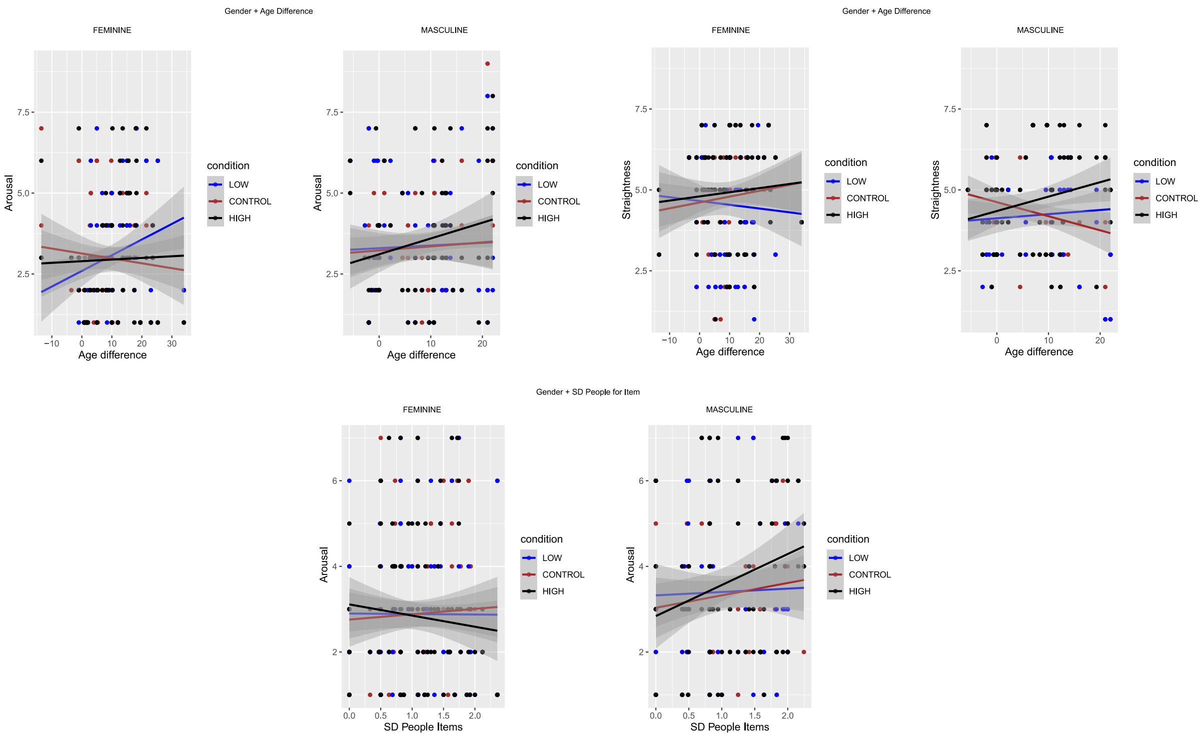

Figure S7: **Interactions with covariates.** Gender. Dots represent the observations, lines show predicted values from the model and shaded regions represent confidence intervals.
